# Supplementary material for: Reversible epigenetic alterations regulate class I HLA loss in prostate cancer
Source: Commun Biol. 2022 Sep 1;5:897. doi: 10.1038/s42003-022-03843-6 (PMC9437063; doi:10.1038/s42003-022-03843-6)
Supplement: Supplementary file 2 — Supplementary Information [file 42003_2022_3843_MOESM2_ESM.pdf]

SUPPLEMENTAL FIGURES

Reversible Epigenetic Alterations Regulate Class I HLA Loss in Prostate Cancer

Tamara S. Rodems, Erika Heninger, Charlotte N. Stahlfeld, Cole S. Gilsdorf, Kristin N. Carlson, Madison R. Kircher, Anupama Singh, Timothy E. G. Krueger, David J. Beebe, David F. Jarrard, Douglas G. McNeel, Michael C. Haffner, and Joshua M. Lang

Figure S1

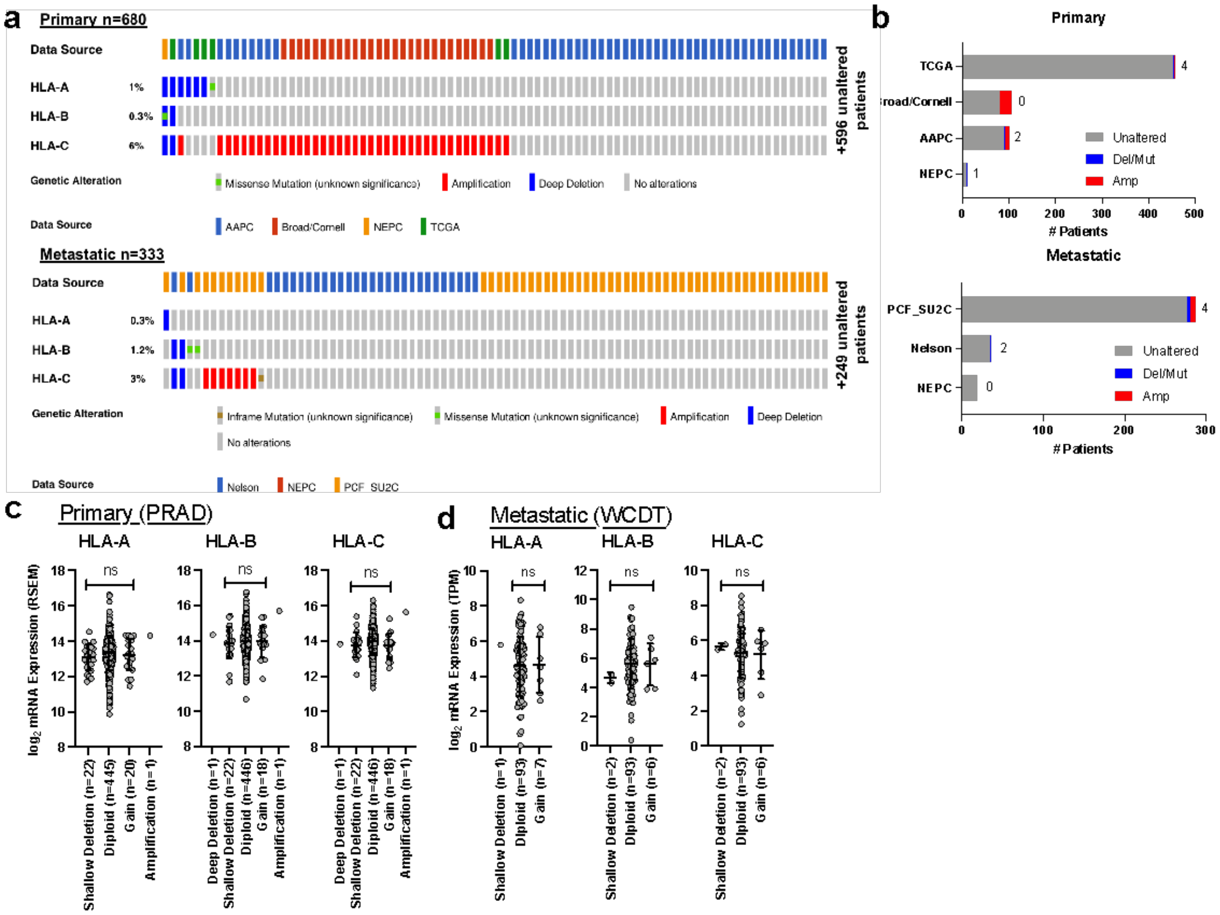

Figure S1. Genomic alterations in primary and metastatic prostate cancer.

(a) Amplifications, deletions, and mutations in HLA-A, HLA-B, and HLA-C in primary prostate adenocarcinoma across 4 independent studies and metastatic prostate adenocarcinoma across 3 independent studies. Overall percent altered for each gene is shown. All existing alterations are shown. **(b)** Data from **(a)** summarized in bar graphs showing number of samples in each study that had each type of alteration in any HLA-I gene. Number of deletion or mutation events is indicated to the right of each bar. **(c)** mRNA expression for primary tumor samples harboring indicated genomic alterations from the TCGA-PRAD (PRAD) data set. **(d)** mRNA expression for metastatic samples harboring indicated genomic alterations from the West Coast Prostate Cancer Dream Team (WCDT) data set.

Figure S2

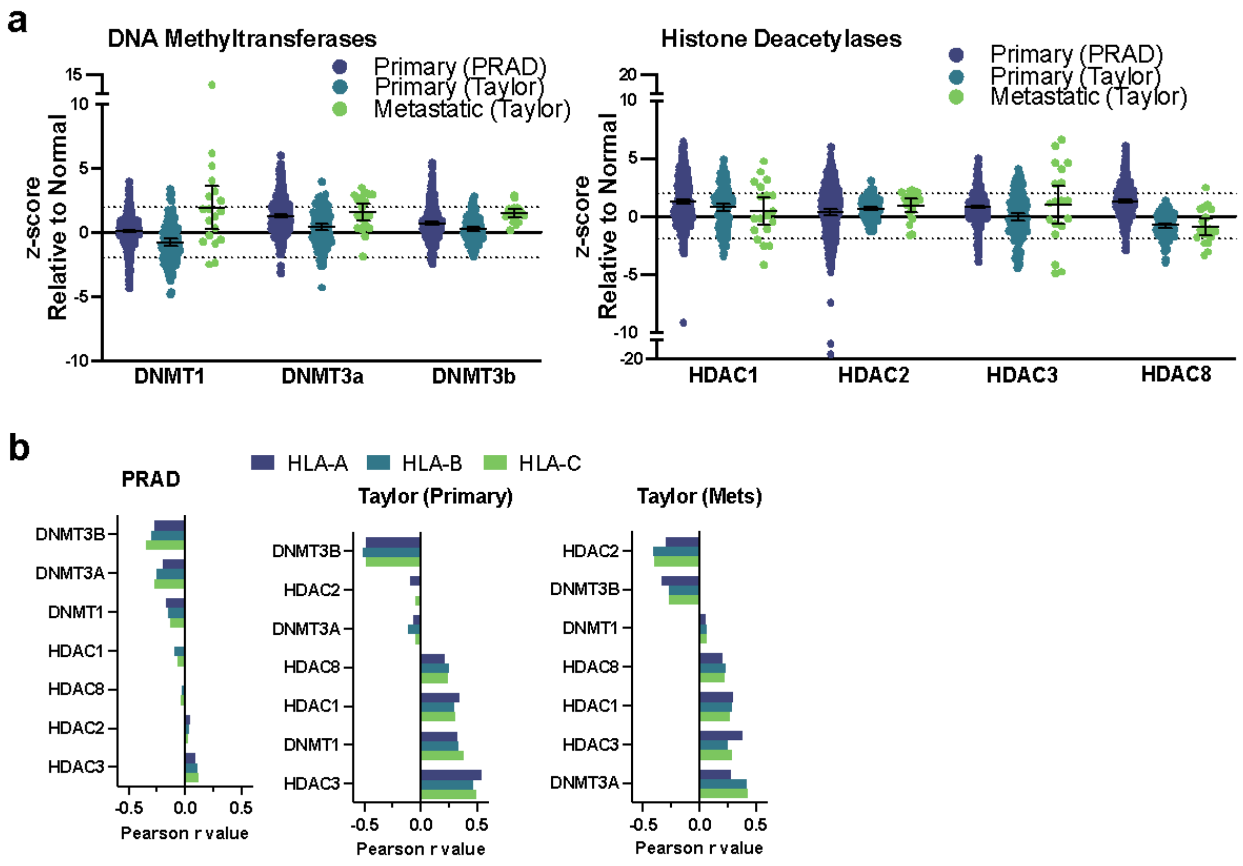

Figure S2. Expression of DNMTs and class I HDACs in prostate cancer.

(a) Expression of selected proteins in the indicated categories of epigenetic modifying proteins in the TCGA-PRAD data set (PRAD, n=497, blue circle), and Taylor data set (Primary n=131, teal circle; Metastatic n=19, green circle). Z-scores are relative to normal samples in the respective study. Line and error bars represent mean and 95% confidence interval. Dotted lines are at +/- 1.96. Expression above or below dotted line is considered significantly up- or downregulated respectively. **(b)** Correlation of expression of epigenetic modifying proteins to HLA-I genes ordered on Pearson's r value.

Figure S3

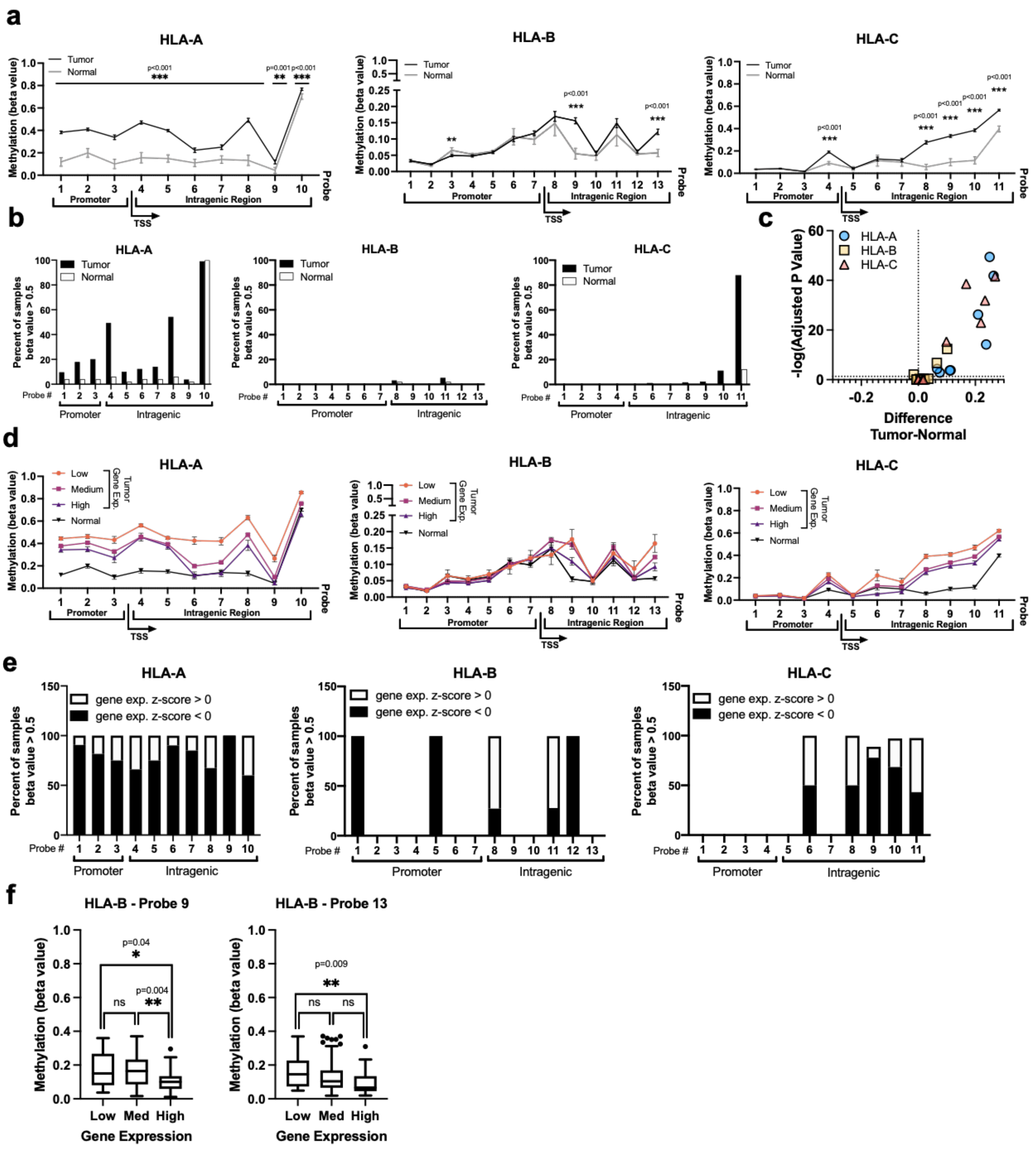

Figure S3. HLA-I DNA methylation stratified by gene expression level.

(a) Methylation levels across the CpG islands of HLA-A (blue circle), HLA-B (yellow square), HLA-C (orange triangle). Gene regions where probes fall, TSS, and CpG island location are annotated. Distances between probes are not to scale. **(b)** Percentage of

tumor and normal samples that had beta values above 0.5 for each probe are shown. Green bar shows probes within the CpG island. Probes within the promoter or intragenic region are also indicated. **(c)** Volcano plot of statistical significance of the difference in methylation levels between tumor and normal samples at each probe. **(d)** Methylation beta values are shown at each probe across the HLA-I promoters and CpG islands for normal samples and tumor samples separated by gene expression level; low(orange circle), medium (pink square), high (purple triangle), normal (black, inverted triangle) . **(e)** Tumor samples with beta values >0.5 for each probe were stratified by gene expression z-score and represented as percentages of total samples with beta values >0.5. **(f)** Box plots of methylation levels of selected HLA-B probes stratified by gene expression level and analyzed by one-way ANOVA. For **(d)** and **(f)** Low expression: z-score < -1.96, medium (med) expression 1.96 < z-score > -1.96, high expression: z-score > 1.96. The number of samples for each probe and within each expression category is available in Table S4.

Figure S4

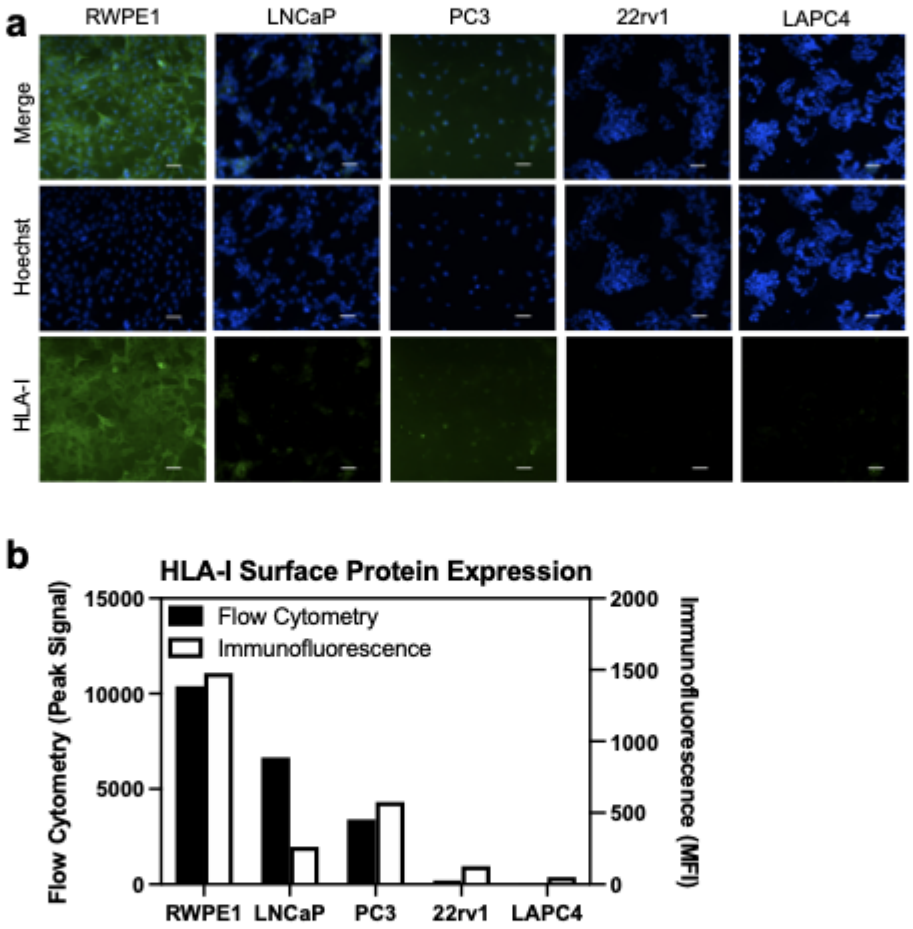

Figure S4. HLA-I protein expression in prostate cancer cell lines.

(a) Extracellular staining of HLA-I visualized by immunofluorescent (IF) microscopy. Scale bars represent 50 μm. **(b)** Representative quantification of flow cytometry and immunofluorescence analysis of HLA-I expression. Data is representative of the histogram in Figure 3B and the IF images in **(a)**.

Figure S5

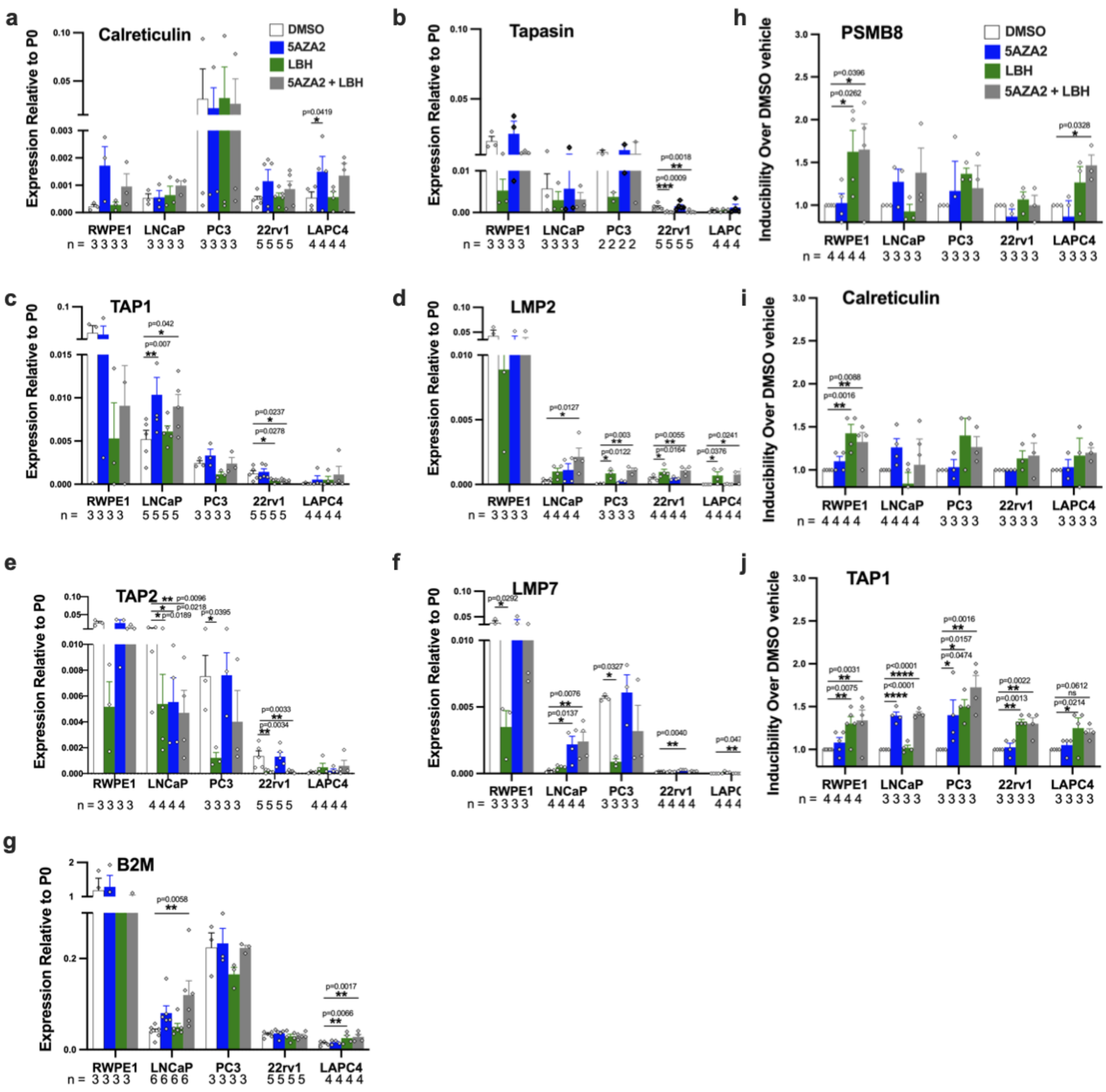

Figure S5. Gene and protein induction of APM and B2M in response to DNMT and HDAC inhibition.

(a-g) Gene expression changes in Calreticulin, Tapasin, *TAP1*, *LMP2*, *TAP2*, *LMP7*, and *B2M* in response to 5AZA2 and LBH alone and in combination. Data show expression relative to housekeeping gene. **(h-j)** Induction of PSMB8, Calreticulin, and TAP1 protein expression in indicated cell lines in response to 5AZA2 and LBH treatment alone and in combination over DMSO vehicle. Error bars represent SEM.

Figure S6

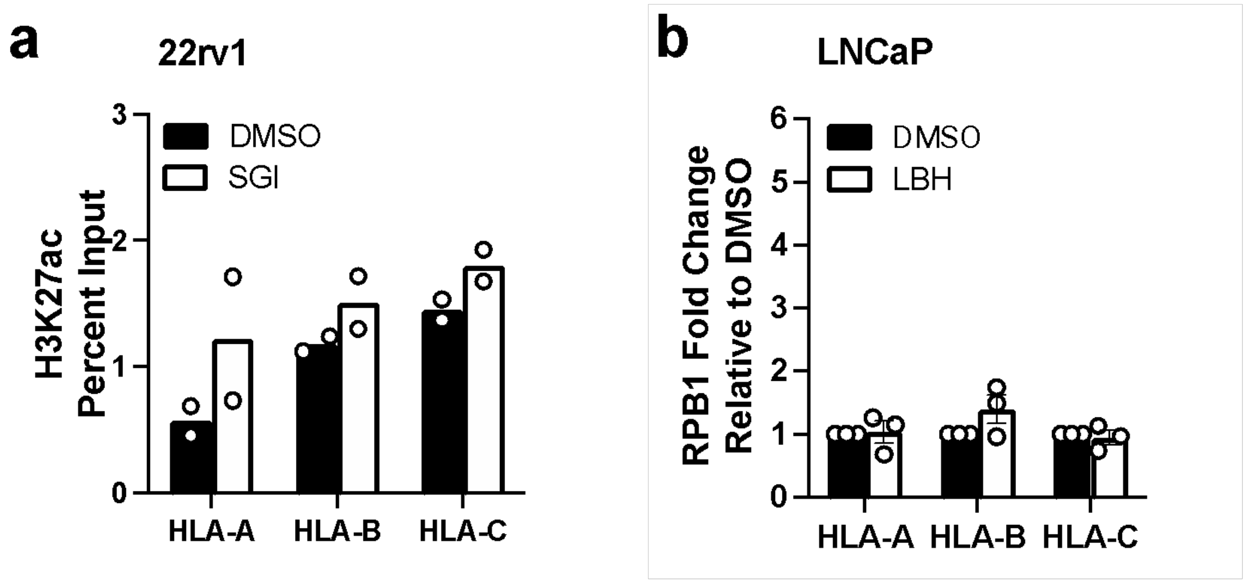

Figure S6. Effect of DNMT inhibition on H3K27ac in 22rv1 cells and effect of HDAC inhibition on RPB1 binding in LNCaP cells.

(a) ChIP using an antibody targeting acetylated lysine 27 on histone H3 (H3K27ac) in 22rv1 cells treated with DMSO or 1uM SGI (n=2). **(b)** ChIP using an antibody targeting RPB1 in LNCaP cells treated with DMSO or 10nM LBH (n=3). Error bars represent SEM.

Figure S7

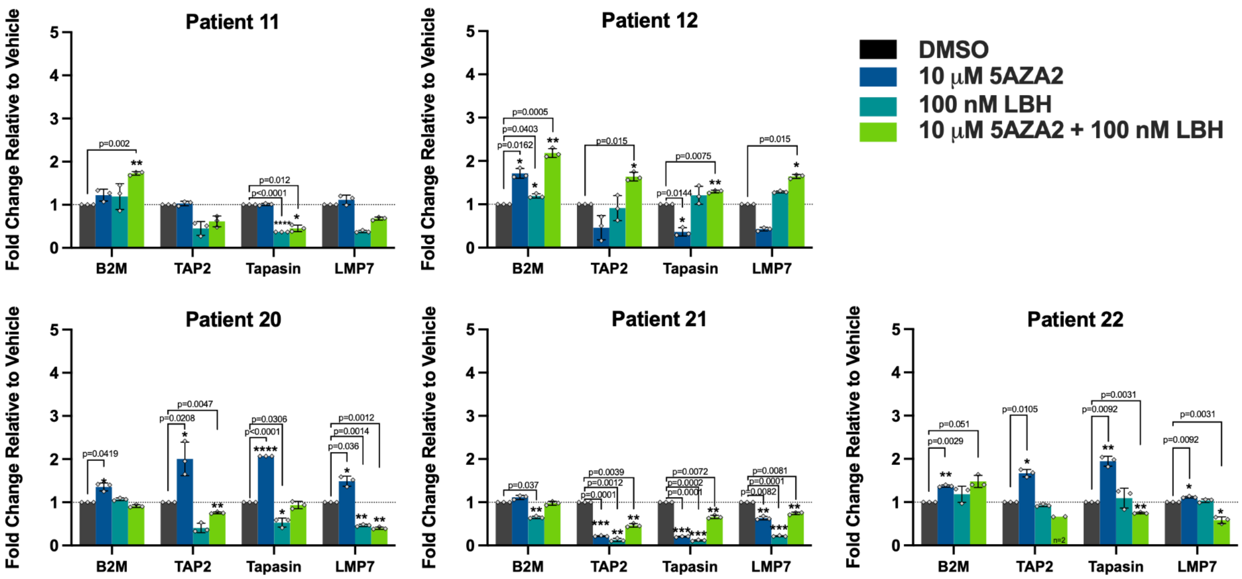

Figure S7. APM and B2M expression in response to DNMT and HDAC inhibition ex vivo.

Gene expression analysis of induction of APM genes and B2M in *ex vivo* tissue treated with DMSO, 5AZA2, LBH, or 5AZA2+LBH. Error bars represent SD; n=3.

Figure S8

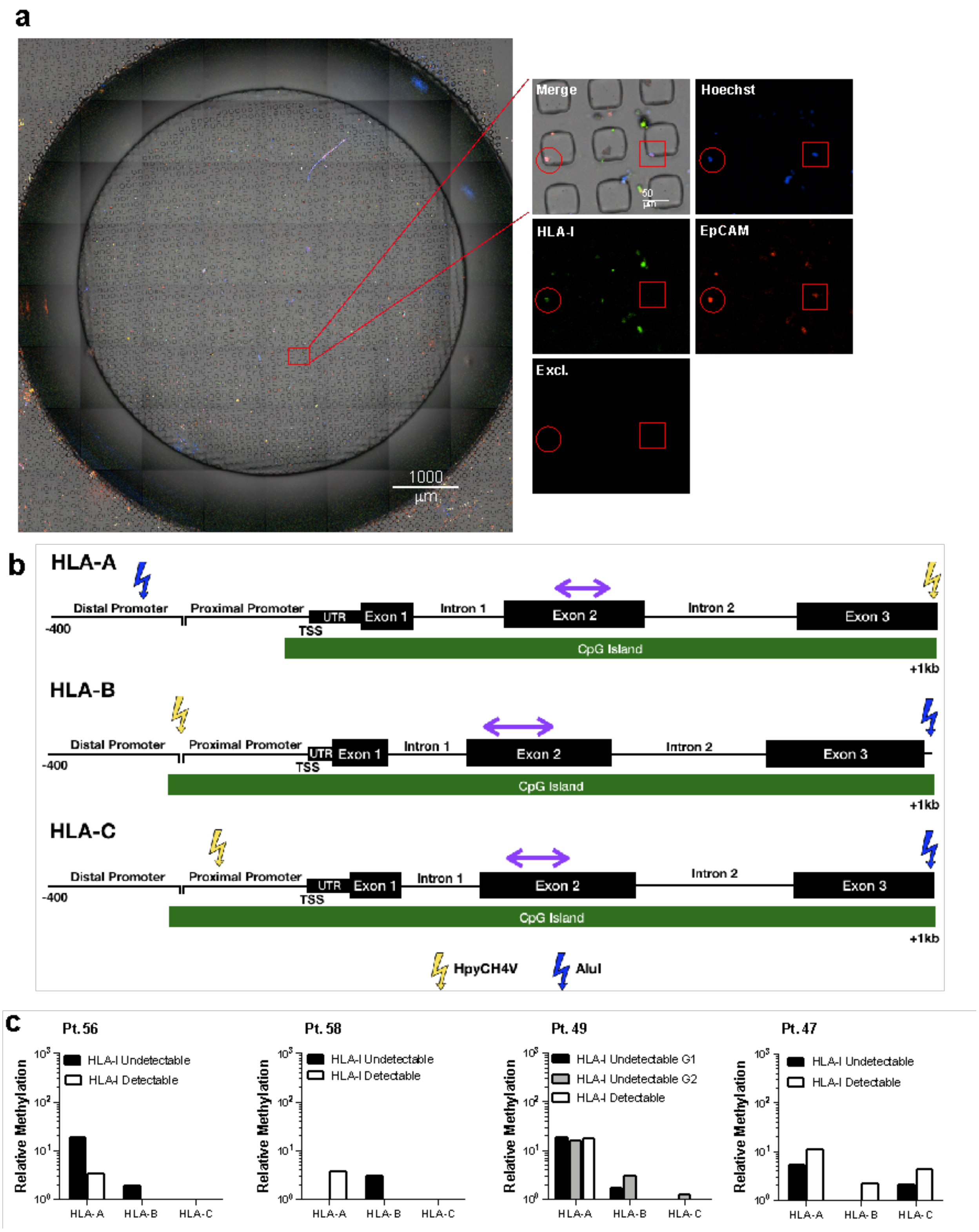
a
1000 µm
Merge
50 µm
Hoechst
HLA-I
EpCAM
Excl.
b
HLA-A
Distal Promoter
Proximal Promoter
UTR
Exon 1
Intron 1
Exon 2
Intron 2
Exon 3
-400
TSS
CpG Island
+1kb
HLA-B
Distal Promoter
Proximal Promoter
UTR
Exon 1
Intron 1
Exon 2
Intron 2
Exon 3
-400
TSS
CpG Island
+1kb
HLA-C
Distal Promoter
Proximal Promoter
UTR
Exon 1
Intron 1
Exon 2
Intron 2
Exon 3
-400
TSS
CpG Island
+1kb
HpyCH4V
AluI
c
Pt. 56
HLA-I Undetectable
HLA-I Detectable
Relative Methylation
HLA-A
HLA-B
HLA-C
Pt. 58
HLA-I Undetectable
HLA-I Detectable
Relative Methylation
HLA-A
HLA-B
HLA-C
Pt. 49
HLA-I Undetectable G1
HLA-I Undetectable G2
HLA-I Detectable
Relative Methylation
HLA-A
HLA-B
HLA-C
Pt. 47
HLA-I Undetectable
HLA-I Detectable
Relative Methylation
HLA-A
HLA-B
HLA-C

Figure S8. Images of seeded microwells used for aspiration of HLA-I positive and negative populations and primer locations.

(a) Image of a seeded microwell (Pt. 56) with zoomed-in images showing one CTC with detectable HLA-I (circle) and one CTC with undetectable HLA-I (red square). **(b)** Primer locations for assessing CTC methylation (purple double-headed arrow). The cut sites of the restriction enzymes used to digest the DNA are indicated (HpyCH4V, yellow jagged arrow; AluI, blue jagged arrow). The fragments used for MBD2-MBD enrichment extend from the first enzyme cut site to the second, with the primer location in the center. **(c)** HLA-I methylation in HLA-I detectable and undetectable groups of approximately 10-15 CTCs represented as methylation relative to detection limit.

Figure S9

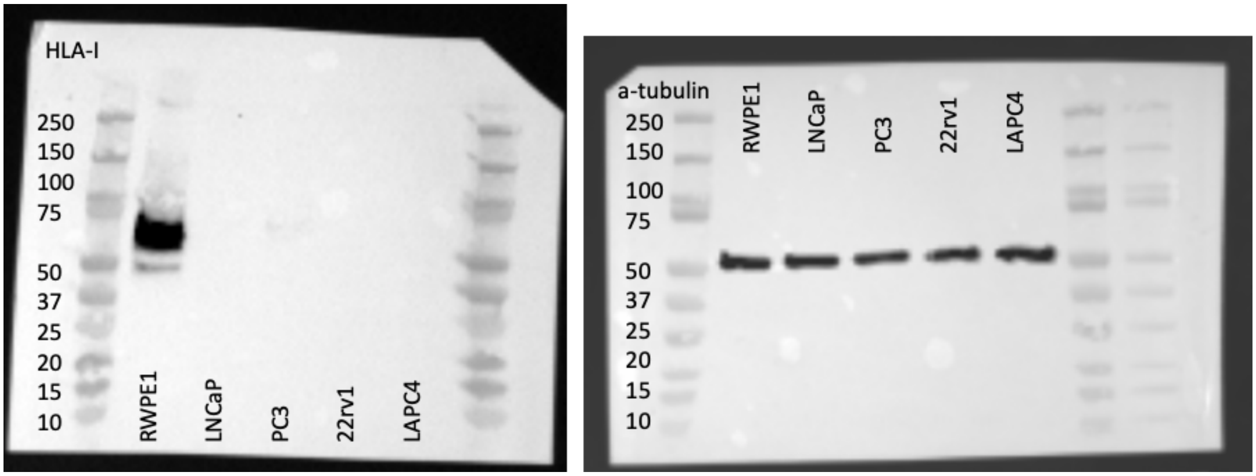

Figure S9. Uncropped, unedited Western blot images for cropped images shown in Figure 3a.

Full blot image for **(a)** HLA-I and **(b)** α-tubulin.
