# Supplementary material for: Reversible epigenetic alterations regulate class I HLA loss in prostate cancer
Source: Commun Biol. 2022 Sep 1;5:897. doi: 10.1038/s42003-022-03843-6 (PMC9437063; doi:10.1038/s42003-022-03843-6)
Supplement: Supplementary file 3 — Description of Additional Supplementary Files [file 42003_2022_3843_MOESM3_ESM.pdf]

Description of Additional Supplementary Files

File name: Supplementary Data 1
Description: Gene expression upregulation and downregulation from public data sets.

File name: Supplementary Data 2
Description: Correlation of HLA to DNMT and HDAC gene expression.

File name: Supplementary Data 3
Description: Correlation of methylation and gene expression from TCGA data.

File name: Supplementary Data 4
Description: TCGA Methylation array probe information.

File name: Supplementary Data 5
Description: Primer information.

File name: Supplementary Data 6
Description: Antibody information.

File name: Supplementary Data 7
Description: Patient characteristics for samples used in single cell aspiration experiments.

File name: Supplementary Data 8
Description: Source data for graphs in the paper.
