# Supplementary material for: Reversible epigenetic alterations regulate class I HLA loss in prostate cancer
Source: Commun Biol. 2022 Sep 1;5:897. doi: 10.1038/s42003-022-03843-6 (PMC9437063; doi:10.1038/s42003-022-03843-6)
Supplement: Supplementary file 12 — Reporting Summary [file 42003_2022_3843_MOESM12_ESM.pdf]

COMMSBIO-21-2961-T
Corresponding author(s): Joshua M Lang

Last updated by author(s): Jul 20, 2022

Reporting Summary

Nature Portfolio wishes to improve the reproducibility of the work that we publish. This form provides structure for consistency and transparency in reporting. For further information on Nature Portfolio policies, see our Editorial Policies and the Editorial Policy Checklist.

Please do not complete any field with "not applicable" or n/a. Refer to the help text for what text to use if an item is not relevant to your study.
For final submission: please carefully check your responses for accuracy; you will not be able to make changes later.

Statistics

For all statistical analyses, confirm that the following items are present in the figure legend, table legend, main text, or Methods section.

n/a	Confirmed	
☐	☒	The exact sample size (*n*) for each experimental group/condition, given as a discrete number and unit of measurement
☐	☒	A statement on whether measurements were taken from distinct samples or whether the same sample was measured repeatedly
☐	☒	The statistical test(s) used AND whether they are one- or two-sided<br>*Only common tests should be described solely by name; describe more complex techniques in the Methods section.*
☐	☒	A description of all covariates tested
☐	☒	A description of any assumptions or corrections, such as tests of normality and adjustment for multiple comparisons
☐	☒	A full description of the statistical parameters including central tendency (e.g. means) or other basic estimates (e.g. regression coefficient) AND variation (e.g. standard deviation) or associated estimates of uncertainty (e.g. confidence intervals)
☐	☒	For null hypothesis testing, the test statistic (e.g. *F*, *t*, *r*) with confidence intervals, effect sizes, degrees of freedom and *P* value noted<br>*Give P values as exact values whenever suitable.*
	☐	For Bayesian analysis, information on the choice of priors and Markov chain Monte Carlo settings
	☐	For hierarchical and complex designs, identification of the appropriate level for tests and full reporting of outcomes
☐	☒	Estimates of effect sizes (e.g. Cohen's *d*, Pearson's *r*), indicating how they were calculated

Our web collection on statistics for biologists contains articles on many of the points above.

Software and code

Policy information about availability of computer code

Data collection: Not applicable

Data analysis: *Provide a description of all commercial, open source and custom code used to analyse the data in this study, specifying the version used OR state that no software was used.*

For manuscripts utilizing custom algorithms or software that are central to the research but not yet described in published literature, software must be made available to editors and reviewers. We strongly encourage code deposition in a community repository (e.g. GitHub). See the Nature Portfolio guidelines for submitting code & software for further information.

Data

Policy information about availability of data

All manuscripts must include a data availability statement. This statement should provide the following information, where applicable:
- Accession codes, unique identifiers, or web links for publicly available datasets
- A description of any restrictions on data availability
- For clinical datasets or third party data, please ensure that the statement adheres to our policy

Data availability statement is included in draft accordingly. Resource data is included in Supplemental Material

Human research participants

Policy information about studies involving human research participants and Sex and Gender in Research.

Reporting on sex and gender	Prostate cancer is present in males only. All the laboratory investigators were blinded to clinical information
Population characteristics	patients were required to have histologically confirmed prostate adenocarcinoma, and documented metastases, as
Recruitment	For prostate tissue donations, patients were consented at the Department of Urology at the School of Medicine and Public Health. For CTC studies, eligible patients were consented by the Biospecimen Disease Oriented Team at the University of Wisconsin, School of Medicine and Public Health.
Ethics oversight	[illegible]

Note that full information on the approval of the study protocol must also be provided in the manuscript.

Field-specific reporting

Please select the one below that is the best fit for your research. If you are not sure, read the appropriate sections before making your selection.

☒ Life sciences ☐ Behavioural & social sciences ☐ Ecological, evolutionary & environmental sciences

For a reference copy of the document with all sections, see nature.com/documents/nr-reporting-summary-flat.pdf

Life sciences study design

All studies must disclose on these points even when the disclosure is negative.

Sample size	
Data exclus	Data points might be excluded in qPCR assays if RPLP0 housekeeping gene was not measurable indicating compromised RNA quality. Data points might be excluded in flow cytometry if sample acquisition was compromised.
Replicatio	
Randomiz	Biospecimens from study subjects and in vitro samples were randomly allocated to experimental treatment groups.
Blinding	Investigators were blinded to clinical data associated with study subjects in experiments including clinical biospecimens. Biospecimens [illegible] this study did not receive any intervention and were not grouped for study purposes.

Behavioural & social sciences study design

All studies must disclose on th

Study description	Not applicable.
Research sample	Not applicable.
Sampling strategy	Not applicable.
Data collection	Not applicable.
Timing	Not applicable.
Data exclusions	Not applicable.
Non-participation	Not applicable.

Randomization Not applicable.

Ecological, evolutionary & environmental sciences study design

All studies must disclose on th[illegible]

Study description	Not applicable.
Research sample	Not applicable.
Sampling strategy	Not [illegible]
Data collection	Not applicable.
Timing and spatial scale	Not applicable.
Data exclusions	Not [illegible]
Reproducibility	Not applicable.
Randomization	Not applicable.
Blinding	Not applicable.

Did the study involve field work? ☐ Yes ☒ No

Field work, collection and transport

Field conditions	*Describe the study conditions for field work, providing relevant parameters (e.g. temperature, rainfall).*
Location	[illegible]
Access & import/export	*[illegible]scribe the efforts you have made to access habitats and to collect and import/export your samples in a responsible manner and in [illegible]npliance with local, national and international laws, noting any permits that were obtained (give the name of the issuing authority, the date of issue, and any identifying information).*
Disturbance	*Describe any disturbance caused by the study and how it was minimized.*

Reporting for specific materials, systems and methods

We require information from authors about some types of materials, experimental systems and methods used in many studies. Here, indicate whether each material, system or method listed is relevant to your study. If you are not sure if a list item applies to your research, read the appropriate section before selecting a response.

Materials & experimental systems

- Antibodies
- Eukaryotic cell lines
- Palaeontology and archaeology
- Animals and other organisms
- Clinical data
- Dual use research of concern

Methods

- ChIP-seq
- Flow cytometry
- MRI-based neuroimaging

Antibodies

Antibodies used

HLA- A,B,C PE W6/32 BioLegend 311406 AB_314875
CD45 Alexa Fluor 647 HI30 BioLegend 304018 AB_389336
CD34 Alexa Fluor 647 581 BioLegend 343508 AB_1877133
CD11b Alexa Fluor 647 ICRF44 BioLegend 301319 AB_493020

ı Cytokeratin* Alexa Fluor 790 C-11 BioLegend 628602 AB_439775

HLA- A,B,C Alexa Fluor 647 W6/32 BioLegend 311414 AB_493135
CD45 PE HI30 BioLegend 304008 AB_314396
)34 PE 581 BioLegend 343506 AB_1731862
CD11b PE M1/70 BioLegend 101208 AB_312791
CD14 PE HCD14 BioLegend 325606 AB_830679
CD27 PE O323 BioLegend 302842 AB_2564146
CD66b PE G10F5 BioLegend 305106 AB_2077857
ı Cytokeratin* Alexa Fluor 790 C-11 BioLegend 628602 AB_439775

HLA- A,B,C PE-Cy7 W6/32 BioLegend 311430 AB_2561617

PSMA 27-38 A02*01 PE pentamer ProImmune F689-2
8a APC-Cy7 53-6.7 Tonbo Biosciences 25-0081 AB_2621623
)69 PerCP H1.2F3 BD Biosciences 551113 AB_394051
anzyme B FITC GB11 BD Biosciences 560211 AB_1645488
I-gamma APC XMG1.2 Tonbo Biosciences 20-7311 AB_2621616
\-1 PE-Cy7 2D7 BD Biosciences 558191 AB_397055
CD107 Brilliant Violet™ 421 1D4B BD Biosciences 564347 AB_2738760

HLA- A,B,C FITC W6/32 BioLegend 311404 AB_314873
CD45 Alexa Fluor 647 HI30 BioLegend 304018 AB_389336
CD34 Alexa Fluor 647 581 BioLegend 343508 AB_1877133
CD11b Alexa Fluor 647 ICRF44 BioLegend 301319 AB_493020
CD27 Alexa Fluor 647 O323 BioLegend 302812 AB_493082
CAM PE VU-1D9 Abcam ab112068 AB_10861805

PSMB8 (LMP7) PE PE EPR14482 Abcam ab210730
Calreticulin Alexa Fluor 647 EPR3924 Abcam ab196159 AB_2819061
P1 FITC TAP1.28 MBL International K0136-4 AB_592808

Validation

Manufacturer's validation, reference checks and internal negative controls were used to validate performance for the follwoing antibodies:
https://www.biolegend.com/fr-ch/products/pe-anti-human-hla-a-b-c-antibody-1872
https://www.biolegend.com/de-at/products/alexa-fluor-647-anti-human-cd45-antibody-2739
https://www.biolegend.com/de-at/products/alexa-fluor-647-anti-human-cd34-antibody-6089
https://www.biolegend.com/de-at/products/alexa-fluor-647-anti-mouse-human-cd11b-antibody-2701
https://www.biolegend.com/de-at/products/purified-anti-cytokeratin-pan-reactive-antibody-2984

https://www.biolegend.com/de-at/products/alexa-fluor-647-anti-human-hla-a-b-c-antibody-2900
https://www.biolegend.com/de-at/products/pe-anti-human-cd45-antibody-708
https://www.biolegend.com/de-at/products/pe-anti-human-cd34-antibody-6033
https://www.biolegend.com/de-at/products/pe-anti-mouse-human-cd11b-antibody-349
https://www.biolegend.com/de-at/products/pe-anti-human-cd14-antibody-3952
https://www.biolegend.com/de-at/products/pe-anti-human-cd27-antibody-811
https://www.biolegend.com/de-at/products/pe-anti-human-cd66b-antibody-6529
https://www.biolegend.com/de-at/products/purified-anti-cytokeratin-pan-reactive-antibody-2984

https://www.biolegend.com/de-at/products/pe-cyanine7-anti-human-hla-a-b-c-antibody-8214

PSMA Tetramer validated by manufacturer ProImmune for each individual batch
https://tonbobio.com/products/apc-cyanine7-anti-mouse-cd8a-53-6-7
https://www.bdbiosciences.com/en-eu/products/reagents/flow-cytometry-reagents/research-reagents/single-color-antibodies-ruo/percp-cy-5-5-hamster-anti-mouse-cd69.551113
https://www.bdbiosciences.com/en-eu/search-results?searchKey=560211
https://tonbobio.com/products/apc-anti-mouse-ifn-gamma-xmg1-2
https://www.bdbiosciences.com/en-eu/search-results?searchKey=558191
https://www.bdbiosciences.com/en-eu/search-results?searchKey=564347

https://www.biolegend.com/de-at/products/fitc-anti-human-hla-a-b-c-antibody-1871
https://www.biolegend.com/de-at/products/alexa-fluor-647-anti-human-cd45-antibody-2739
https://www.biolegend.com/de-at/products/alexa-fluor-647-anti-human-cd34-antibody-6089
https://www.biolegend.com/de-at/products/alexa-fluor-647-anti-human-cd11b-antibody-3248
https://www.biolegend.com/de-at/products/alexa-fluor-647-anti-human-cd27-antibody-3271
https://www.abcam.com/pe-epcam-antibody-vu-1d9-ab112068.html

https://www.abcam.com/pe-proteasome-20s-lmp7-antibody-epr14482b-ab210730.html
https://www.abcam.com/alexa-fluor-647-calreticulin-antibody-epr3924-er-marker-ab196159.html
https://www.mblintl.com/products/k0136-4/

Eukaryotic cell lines

Policy information about cell lines and Sex and Gender in Research

Cell line source(s)	LAPC4 (ATCC, Cat# CRL-13009, RRID: CVCL_4744) RWPE1 (ATCC Cat# CRL-11609, RRID: CVCL_3791, LNCaP (ATCC, Cat# CRL-1740, RRID: CVCL_1379), 22Rv1 (ATCC, Cat# CRL-2505, RRID: CVCL_1045), PC3 (ATCC, Cat# CRL-1435, RRID: CVCL_0035) LCL (HCC2218-BL, ATCC, Cat# CRL-2363, RRID: CVCL_1264)
Authentication	Cell lines were authenticated by short tandem repeat at the TRIP Laboratory at the Department of Pathology, University of Wisconsin.
Mycoplasma contamination	Cell lines were mycoplasma by PCR in 2017 at the TRIP Laboratory at the Department of Pathology, University of Wisconsin.
Commonly misidentified lines (See ICLAC register)	No commonly misidentified cell lines were used in this study.

Palaeontology and Archaeology

Specimen provenance	*Provide provenance information for specimens and describe permits that were obtained for the work (including the name of the issuing authority, the date of issue, and any identifying information). Permits should encompass collection and, where applicable, export.*
Specimen deposition	*Indicate where the specimens have been deposited to permit free access by other researchers*
Dating methods	*If new dates are provided, describe how they were obtained (e.g. collection, storage, sample pretreatment and measurement), where they were obtained (i.e. lab name), the calibration program and the protocol for quality assurance OR state that no new dates are provided.*

☐ Tick this box to confirm that the raw and calibrated dates are available in the paper or in Supplementary Information.

Ethics oversight	*Identify the organization(s) that approved or provided guidance on the study protocol, OR state that no ethical approval or guidance was required and explain why not.*

Note that full information on the approval of the study protocol must also be provided in the manuscript.

Animals and other research organisms

Policy information about studies involving animals; ARRIVE guidelines recommended for reporting animal research, and Sex and Gender in Research

Laboratory animals	Transgenic HHD mice were utilized in this study.
Wild animals	No wild animals were used in this study.
Reporting on sex	Research included random selection of male and female animals. Sex based data disaggregation has not been performed and recorded. Murine immune cells were utilized in this study as sentinel agents only and sex-based differences were not assessed in study design.
Field-collected samples	No field collected samples were used in this study.
Ethics oversight	Research animals were maintained and experiments were performed in accordance with institutional guidelines overseen by the Institutional Animal Care and Use Comittee of the University of Wisconsin.

Note that full information on the approval of the study protocol must also be provided in the manuscript.

Clinical data

Policy information about clinical studies

All manuscripts should comply with the ICMJE guidelines for publication of clinical research and a completed CONSORT checklist must be included with all submissions.

Clinical trial registration	Not applicable.
Study protocol	Not applicable.
Data collection	All the laboratory investigators were blinded to clinical information in this study
Outcomes	Not applicable.

Dual use research of concern

Policy information about [dual use research of concern](#)

Hazards

Could the accidental, deliberate or reckless misuse of agents or technologies generated in the work, or the application of information presented in the manuscript, pose a threat to:

No Yes

[redacted]blic health
[redacted]tional security
[redacted]ops and/or livestock
[redacted]osystems
[redacted]y other significant area

Experiments of concern

Does the work involve any of these experiments of concern:

No Yes

[redacted]monstrate how to render a vaccine ineffective
[redacted]nfer resistance to therapeutically useful antibiotics or antiviral agents
[redacted]hance the virulence of a pathogen or render a nonpathogen virulent
[redacted]rease transmissibility of a pathogen
[redacted]er the host range of a pathogen
[redacted]able evasion of diagnostic/detection modalities
[redacted]able the weaponization of a biological agent or toxin
[redacted]y other potentially harmful combination of experiments and agents

[redacted]hIP-seq

Data deposition

☐ Confirm that both raw and final processed data have been deposited in a public database such as [GEO](#).

☐ Confirm that you have deposited or provided access to graph files (e.g. BED files) for the called peaks.

Data access links<br>*May remain private before publication.*	*For "Initial submission" or "Revised version" documents, provide reviewer access links. For your "Final submission" document, provide a link to the deposited data.*
Files in database submission	*Provide a list of all files available in the database submission.*
Genome browser session<br>(e.g. [UCSC](#))	[illegible]

Methodology

Replicates	*Describe the experimental replicates, specifying number, type and replicate agreement*
Sequencing depth	*wh*[illegible]
Antibodies	*[illegible]scribe the antibodies used for the ChIP-seq experiments; as applicable, provide supplier name, catalog number, clone name, and lot [illegible]mber.*
Peak calling parameters	
Data quality	*Describe the methods used to ensure data quality in full detail, including how many peaks are at FDR 5% and above 5-fold enrichment.*
Software	*[illegible] repository, provide accession details.*

Flow Cytometry

Plots

Confirm that:

☒ The axis labels state the marker and fluorochrome used (e.g. CD4-FITC).

☒ The axis scales are clearly visible. Include numbers along axes only for bottom left plot of group (a 'group' is an analysis of identical markers).

☒ All plots are contour plots with outliers or pseudocolor plots.

☒ A numerical value for number of cells or percentage (with statistics) is provided.

Methodology

Sample preparation	murine splenocytes generated.
Instrument	
Software	FlowJo software v9.9.6 (FlowJo, RRID: SCR_008520)
Cell population abundance	Statistics show frequency within parent gates defined in figure legend
Gating strategy	Gating strategy is pictured in draft and described in methods and figure legends.

☐ Tick this box to confirm that a figure exemplifying the gating strategy is provided in the Supplementary Information.

Magnetic resonance imaging

Experimental design

Design type	*Indicate task or resting state; event-related or block design.*
Design specifications	
Behavioral performance mea[illegible]	*State number and/or type of variables recorded (e.g. correct button press, response time) and what statistics were used to establish that the subjects were performing the task as expected (e.g. mean, range, and/or standard deviation across subjects).*

Acquisition

Imaging type(s)	*Specify: functional, structural, diffusion, perfusion.*
Field strength	*Specify in Tesla*
Sequence & imaging parameters	*Specify the pulse sequence type (gradient echo, spin echo, etc.), imaging type (EPI, spiral, etc.), field of view, matrix size,*
Area of acquisition	*State whether a whole brain scan was used OR define the area of acquisition, describing how the region was determined.*
Diffusion MRI	☐ Use[illegible]

Preprocessing

Preprocessing software	*Provide detail on software version and revision number and on specific parameters (model/functions, brain extraction, segmentation, smoothing kernel size, etc.).*
Normalization	
Normalization template	*original Talairach, MNI305, ICBM152) OR indicate that the data were not normalized.*
Noise and artifact removal	*physiological signals (heart rate, respiration).*
Volume censoring	*Define your software and/or method and criteria for volume censoring, and state the extent of such censoring*

Statistical modeling & inference

Model type and settings	*Specify type (mass univariate, multivariate, RSA, predictive, etc.) and describe essential details of the model at the first and second levels (e.g. fixed, random or mixed effects; drift or auto-correlation).*
Effect(s) tested	*Define precise effect in terms of the task or stimulus conditions instead of psychological concepts and indicate whether ANOVA or factorial designs were used.*

Specify type of analysis: ☐ Whole brain ☐ ROI-based ☐ Both

Statistic type for inference (See Eklund et al. 2016)	*Specify voxel-wise or cluster-wise and report all relevant parameters for cluster-wise methods.*
Correction	*Describe the type of correction and how it is obtained for multiple comparisons (e.g. FWE, FDR, permutation or Monte Carlo).*

Models & analysis

n/a	Involved in the study
☐	☐ Functional and/or effective connectivity
☐	☐ Graph analysis
☐	☐ Multivariate modeling or predictive analysis

Functional and/or effective connectivity	*Report the measures of dependence used and the model details (e.g. Pearson correlation, partial correlation, mutual information).*
Graph analysis	*Report the dependent variable and connectivity measure, specifying weighted graph or binarized graph, subject- or group-level, and the global and/or node summaries used (e.g. clustering coefficient, efficiency, etc.).*
Multivariate modeling and predictive analysis	*Specify independent variables, features extraction and dimension reduction, model, training and evaluation metrics.*

his checklist template is licensed under a Creative Commons Attribution 4.0 International License, which permits use, sharing, adaptation, distribution and reproduction in any medium or format, as long as you give ppropriate credit to the original author(s) and the source, provide a link to the Creative Commons license, and indicate if changes were made. The images or other third party material in this article are included in ie article's Creative Commons license, unless indicated otherwise in a credit line to the material. If material is not included in the article's Creative Commons license and your intended use is not permitted by :atutory regulation or exceeds the permitted use, you will need to obtain permission directly from the copyright holder. To view a copy of this license, visit http://creativecommons.org/licenses/by/4.0/
